# Supplementary material for: Bovine brucellosis seropositivity in Mpumalanga Province, South Africa, 2021–2024: Temporal, and spatial trends
Source: PLoS One. 2026 Mar 6;21(3):e0344037. doi: 10.1371/journal.pone.0344037 (PMC12965600; doi:10.1371/journal.pone.0344037)
Supplement: Attachment — Submitted filename: Response to reviewers.docx [file pone.0344037.s002.docx]

**Reviewer #1**

**General assessment**

**Reviewer comment**: The manuscript is well written, methodologically sound, and addresses an important knowledge gap. The conclusions are supported by the data and relevant to national and international brucellosis control strategies.

**Authours’ response**: We sincerely thank Reviewer #1 for the positive evaluation of the manuscript and for recognising its relevance and methodological rigour. No changes were required in response to this overall assessment.

__________________________________________________________________________________

**Methods**

**Reviewer comment**: The methodology is clear, ethical approval is appropriate, laboratory procedures follow WOAH guidelines, and statistical methods are sufficiently described.

**Authours’ response**: We appreciate this positive assessment. In response to comments from other reviewers, we have further expanded the Methods section to improve reproducibility by explicitly describing data inclusion/exclusion criteria, data cleaning procedures, variable coding, regression model specification, and diagnostic evaluation.

__________________________________________________________________________________

**Results**

**Reviewer comment**: An inconsistency was noted between Table 3 and the text regarding the ranking of Emalahleni and Victor Khanye.

**Authours’ response**: Thank you for highlighting this inconsistency. The text has been corrected to accurately reflect Table 3, confirming that Emalahleni recorded the highest seroprevalence, followed by Victor Khanye. Consistency between tables and narrative has been verified throughout the Results section.

__________________________________________________________________________________

**Reviewer #2**

**Introduction**

**Reviewer comment**: The introduction is comprehensive, current, and well aligned with the objectives. However, justification for the 2021–2024 study period is missing, and links to national control programmes could be strengthened.

**Authours’ response**: We have revised the final paragraph of the Introduction to explicitly justify the 2021–2024 period, linking it to intensified provincial surveillance activities and national brucellosis control efforts, including vaccination and routine laboratory monitoring. Additional context on socioeconomic implications has also been incorporated.

___________________________________________________________________

**Methods**

**Reviewer comment**: Clarify whether confirmatory testing (CFT) was used and consider adding a statement on replicability (e.g., Stata code availability).

**Authours’ response**: We have clarified that routine confirmatory testing (CFT/ELISA) was not systematically available for all records included in this retrospective dataset and that RBT results were therefore analysed as presumptive positives. A clear statement on replicability has been added, indicating that aggregated data and analysis code are available through a public repository, subject to data-sharing regulations.

___________________________________________________________________

**Statistical analysis**

**Reviewer comment**: Reviewer comment

Seasonal effects show unexpected directionality in the adjusted model. Please comment on possible multicollinearity or sampling patterns and confirm sample sizes per season.

**Authours’ response**: We have revised the Results and Discussion sections to clarify that changes in seasonal associations after adjustment likely reflect correlations between year, season, and submission patterns, rather than biological reversal of risk. Seasonal sample sizes used in adjusted models are now explicitly reported, and interpretations have been appropriately tempered.

___________________________________________________________________

**Results and tables**

**Reviewer comment**: Some table footnotes lack abbreviation definitions, and Table 5 seasonal findings require clearer explanation.

**Authours’ response**: All tables have been revised to include complete footnotes defining abbreviations (e.g., CI, OR, AOR). The Results text now explicitly states which predictors were statistically significant before and after adjustment, with clearer explanation of seasonal findings in Table 5.

___________________________________________________________________

**Reviewer #3**

**General assessment**

**Reviewer comment**: The topic is important, but major revisions are required due to internal inconsistencies, insufficient methodological detail, limited spatial analysis, and reference issues.

**Authours’ response**: We thank Reviewer #3 for this detailed critique. The manuscript has undergone substantial revision to address all major concerns, as detailed below.

__________________________________________________________________

**Methods and reproducibility**

**Reviewer comment**: Key variables, reference categories, data cleaning procedures, season derivation, LMA coding, clustering considerations, and model diagnostics are insufficiently described.

**Authours’ response**: The Methods section has been comprehensively revised to:

- Explicitly define the dependent and independent variables, categories, and reference groups
- Describe inclusion/exclusion criteria, handling of missing data, duplicate batch removal, and consistency checks
- Clarify that seasons were derived from sample receipt dates
- Specify LMA coding and justify the absence of multilevel modelling due to lack of herd-level identifiers
- Report numerical results for model diagnostics (Hosmer–Lemeshow test and AUC)

___________________________________________________________________

**Statistical inconsistencies**

**Reviewer comment**: There is inconsistency between stated reference seasons and Table 5, suggesting coding or reporting errors.

**Authours’ response**: Thank you for identifying this issue. The regression models were re-checked, and the reference categories have been standardised and explicitly stated in both the Methods and Tables. Table 5 has been corrected, and all interpretations in the Results and Discussion now align with the final model specification.

___________________________________________________________________

**Spatial analysis**

Reviewer comment: Spatial patterns are presented only in tables; a map or spatial visualisation is needed to justify “hotspot” claims.

**Authours’ response**: We agree with this recommendation. A choropleth map of LMA-level seroprevalence has been added to visually depict spatial heterogeneity. In addition, language referring to “hotspots” has been moderated to reflect descriptive rather than inferential spatial analysis.

___________________________________________________________________

**Discussion**

**Reviewer comment**: Some explanations are speculative, spatial hotspot claims are overstated, and the discussion is repetitive.

**Authours’ response**: The Discussion has been revised to:

- Clearly label explanations related to vaccination, movement, and biosecurity as hypotheses
- Temper claims regarding spatial hotspots
- Reduce repetition of numerical results and focus more on interpretation
- Integrate limitations into the final paragraph of the Discussion, without a separate subheading

___________________________________________________________________

**Strengths and limitations**

**Reviewer comment**: Limitations related to routine laboratory data quality and lack of spatial visualisation are not sufficiently acknowledged.

**Authours’ response**: These limitations are now explicitly acknowledged in the final paragraph of the Discussion, including data incompleteness, potential duplicate submissions, absence of herd-level metadata, and limited spatial modelling. Capitalisation and sentence structure have also been corrected.

___________________________________________________________________

**Conclusion**

**Reviewer comment**: Some statements imply causality and should be presented more cautiously.

**Authours’ response**: The Conclusion has been revised to avoid causal language and to emphasise that findings represent associations from a retrospective observational study. Seasonal and spatial interpretations are now more cautious and aligned with the analytical evidence.

___________________________________________________________________

**References**

**Reviewer comment**: Several references are outdated, incomplete, or incorrect.

**Authours’ response**: The reference list has been thoroughly reviewed. Incorrect or unverifiable references have been corrected or removed, DOIs updated where available, and recent literature prioritised to ensure accuracy and relevance.

___________________________________________________________________
