# Supplementary material for: Bovine brucellosis seropositivity in Mpumalanga Province, South Africa, 2021–2024: Temporal, and spatial trends
Source: PLoS One. 2026 Mar 6;21(3):e0344037. doi: 10.1371/journal.pone.0344037 (PMC12965600; doi:10.1371/journal.pone.0344037)
Supplement: Attachment — Submitted filename: Response to Reviewer revised.docx [file pone.0344037.s003.docx]

**Response to Reviewers**

We sincerely thank the Editor and the reviewers for their careful evaluation of the manuscript and for their constructive comments. We appreciate the positive assessment of the revised version and have addressed the remaining minor issues as requested. All changes have been incorporated into the manuscript, and the reference list has been thoroughly revised.

**Reviewer 1**

**Comment**: References are important in research as they provide evidence, facilitate exploration of sources, and establish credibility. However, the comments regarding the reference section in the initial review were overlooked. Minor revisions are recommended to address these issues.

**Response**: We thank the reviewer for highlighting the importance of the reference section. The references have now been comprehensively revised to ensure full compliance with the journal’s formatting and citation requirements.

**Revisions made**:

- All references have been renumbered sequentially according to their first appearance in the text.
- In-text citations have been cross-checked to ensure one-to-one correspondence with the reference list.
- Formatting has been standardised to the journal’s required style.
- Recent literature has been prioritised where available, while older foundational references have been retained only where necessary for historical or methodological context.

**Reviewer 2**

**Comment**: The reviewer commends the substantial improvements and supports publication, with no further substantive comments.

**Response**: We thank the reviewer for the positive evaluation of the revised manuscript and for acknowledging the improvements in methodological transparency, statistical reporting, and interpretation. No further changes were required in response to this review.

**Reviewer 3**

**Comment 1**: Reference formatting and numbering - Reference formatting remains inconsistent. Citations are not numbered and ordered consistently, and some in-text citations do not correspond clearly to the reference list. The authors should renumber all references sequentially and ensure formatting compliance.

**Response**: We appreciate this observation and have performed a full audit of the reference section.

**Revisions made**:

- All references were renumbered in sequential order according to first citation.
- In-text citations were cross-checked line by line against the reference list.
- Duplicate, mismatched, or unclear citations were corrected.
- Formatting was standardised according to the journal’s reference style.

**Comment 2**: Use of recent literature - Several cited sources appear to predate the last five years. The authors should ensure that the majority of references reflect recent literature and justify older sources.

**Response**: We have updated the reference list to improve representation of recent literature.

**Revisions made**:

Recent studies (2020–2024) were incorporated where relevant, particularly for:

- One Health frameworks
- Surveillance strategies
- Spatial epidemiology
- Control programme evaluations
- Older references were retained only where they represent:
- Foundational epidemiological evidence
- Key diagnostic or control programme references
- Legislative or policy sources

**Comment 3**: Strengths and limitations section - The manuscript lacks a clearly outlined Strengths and Limitations section. A dedicated subsection is required.

**Response**: We agree with the reviewer. A new subsection titled “Strengths and Limitations” has been added to the Discussion to improve transparency and interpretation.
