# Supplementary material for: Bovine brucellosis seropositivity in Mpumalanga Province, South Africa, 2021–2024: Temporal, and spatial trends
Source: PLoS One. 2026 Mar 6;21(3):e0344037. doi: 10.1371/journal.pone.0344037 (PMC12965600; doi:10.1371/journal.pone.0344037)
Supplement: Attachment — Submitted filename: Rebuttal letter 120226.docx [file pone.0344037.s004.docx]

Dear Editor,

We thank you for the careful evaluation of our manuscript and for the constructive guidance provided. We have addressed all journal and editorial requirements and revised the manuscript accordingly. A detailed, point-by-point response is provided below.

**Journal Requirement 1**

**Comment**: If the reviewer comments include a recommendation to cite specific previously published works, please review and evaluate these publications to determine whether they are relevant and should be cited.

**Response**: We carefully reviewed all reviewer comments and evaluated the relevance of any suggested references. Where suggested works were directly relevant to the scope, methodology, or interpretation of our findings, they were incorporated into the manuscript. References that were not directly relevant were not included, in accordance with the journal’s guidance that citation of suggested works is not mandatory unless instructed by the editor.

**Journal Requirement 2**

**Comment**: Please review your reference list to ensure that it is complete and correct. If you have cited papers that have been retracted, please include the rationale for doing so or remove them.

**Response**: The entire reference list was carefully reviewed to ensure:

- Accuracy of bibliographic information
- Correct and functional DOIs
- Consistency with journal formatting
- Relevance to the manuscript

A check was conducted to identify any retracted publications. No retracted articles were identified in the reference list. Therefore, no references were removed or replaced on the basis of retraction, and no additional explanatory statements were required in the manuscript. Minor corrections to reference formatting and consistency were implemented where necessary. These changes are reflected in the revised manuscript.

**Additional Editor Comment**

**Comment**: Please remove all subheadings from the Discussion section, including “Strengths and Limitations of the Study.” The study limitations should be presented as the final paragraph of the Discussion.

**Response**: We have revised the Discussion section as requested:

- All subheadings have been removed from the Discussion section, including the subheading “Strengths and limitations of the study.”
- The content previously presented under this subheading has been integrated into the main Discussion text. The study limitations are now presented as the final paragraph of the Discussion, in narrative form.
